# Awake tracheal intubation in routine airway management: A retrospective analysis in a tertiary centre

**DOI:** 10.1371/journal.pone.0299071

**Published:** 2024-03-01

**Authors:** Nina Pirlich, Alexander Berk, Regina Hummel, Irene Schmidtmann, Katharina Epp, Marc Kriege, Eva Wittenmeier

**Affiliations:** 1 Department of Anesthesiology, University Medical Centre of the Johannes Gutenberg-University Mainz, Mainz, Germany; 2 Institute of Medical Biostatistics, Epidemiology and Informatics, University Medical Centre of the Johannes Gutenberg-University Mainz, Mainz, Germany; Sapienza University of Rome: Universita degli Studi di Roma La Sapienza, ITALY

## Abstract

**Introduction:**

While awake tracheal intubation (ATI) is regarded as the gold standard for difficult airway management according to current guidelines, there seems to be a reluctance in its application. This retrospective cohort study, conducted at a German tertiary hospital over a 2-year period, aimed to demonstrate that integrating awake tracheal intubation using flexible bronchoscopy (ATI:FB) into routine airway management makes it a successful and safe approach.

**Materials and methods:**

In 2019 and 2020, records from the data acquisition system (DAQ) and archived anesthesia records were screened to evaluate the specifics of ATI:FB procedures, focusing on overall success and safety. Analysis included complications, time required for ATI:FB, and potential influencing factors such as patient characteristics, indication, medical/operative specialty, sedation technique, route and experience of anesthesiologist. Logistic regression assessed the impact of various variables on occurrence of complications and linear regression, with log(time) as the dependent variable, evaluated median time required to perform ATI:FB.

**Results:**

ATI:FB constituted 4.3 % (n = 1,911) of all airway management procedures, predominantly observed in dental, oral, and maxillofacial surgery (46.5 %) and otorhinolaryngology (38.4 %). The success rate for ATI:FB was notably high at 99.6 %, with only 5.4 % of cases experiencing complications, including technical issues, agitation, and visibility obstruction due to mucous secretion. Complication risk was influenced by the medical specialty and the experience of the anesthesiologist. A strong effect was observed in otorhinolaryngology (OR = 4.54, 95 % CI [1.64; 14.06]). The median time required for ATI:FB was 16 minutes (IQR: 11 to 23), with factors such as indication (p < 0.0001), experience of anesthesiologist (p < 0.0001), sedation technique (p = 0.0408), priority of the procedure (p = 0.0134), and medical/operative specialty (p < 0.0001) affecting the duration. The median time required for ATI:FB differed significantly based on the experience of the anesthesiologist (p < 0.0001).

**Conclusion:**

ATI:FB proves successful and safe, with low complications and manageable procedural time. Experience of the anesthesiologist is a modifiable factor enhancing safety, emphasizing the need for ATI:FB integration into routine airway management.

## Introduction

The first practice guideline on awake tracheal intubation (ATI) was published by the Difficult Airway Society (DAS) in 2020 [1]. Despite the high success rate and a favorable safety profile associated of ATI; this essential technique may be underused for anticipated difficult airway management [1]. Other national data on the use of ATI is largely lacking [2], with available data reported only for single tertiary institutions in Canada, the USA and the UK [3–6].

ATI is most-commonly conducted using flexible bronchoscopy (ATI:FB) in accordance with current recommendations. Our recent study on the frequency of ATI in Germany revealed a discrepancy between the availability of ATI:FB equipment for German anesthesiologists (> 90 %) and their proficiency in the technique (60 %) [7]. Only half of German anesthesiologists stated that they had performed more than 25 ATI:FBs throughout their career. This reluctance in performing ATI may be attributed to a fear of discomfort to the awake patient and poor patient cooperation, a lack of ATI practice training, and the availability of alternative techniques used after the induction of general anesthesia [6]. ATI may also be perceived as time-consuming and complicated, and hence, anesthesiologists may particularly favor video laryngoscopy after the induction of anesthesia. It is noteworthy to mention at this point that video laryngoscopy performed as an awake tracheal intubation (ATI:VL) is an alternative method to ATI:FB when the presenting anatomy permits, and with sufficient clinician experience. ATI:VL is advantageous for preserving airways in severe physiologic derangements within critical care or emergency medicine, but contraindications include certain anatomical presentations, such as limited mouth opening. However, these concerns are offset by the major potential safety benefits of ATI. During a correctly performed ATI the airway remains continuously open, and the patient breathes by himself, making hypoxia much less likely [8]. Therefore, ATI is also recommended in the German S1 guideline for airway management for the anticipated difficult airway. The recommendation entails the use of a flexible intubation endoscope (ATI:FB) and stresses the importance of training and practice in this technique [9]. In our lately published large scale analysis over a 16-year period, 5.5 % of all airway procedures in our high volume tertiary centre were ATI:FBs [10]. This proportion is comparatively high in light of previously reported data [3, 5, 6] Considering ATI as a routine component in our airway management, we conducted now a retrospective study with the aim of contributing more detailed data on ATI from Germany to the international airway community for the first time.

The main objective of our retrospective cohort study was to evaluate the overall success of ATI at our tertiary center over a 2-year period. The secondary outcome focused on assessing the safety profile of ATI. Safety was defined through an analysis of complications and the time required for the procedure. Complications and prolonged procedure duration present potential risks that could compromise patient safety. Therefore, we examined complications, the time needed for ATI, and factors such as indication, patient characteristics, and experience of the anesthesiologist to identify and understand influencing factors on safety.

Our hypothesis posits that ATI, when integrated into routine airway management, represents a successful and safe method for managing the expected difficult airway.

## Materials and methods

The study was performed in accordance with the Strengthening the Reporting of Observational Studies in Epidemiology (STROBE) recommendations [11] and approved in September 2020 by the Ethics Committee of the Medical Association of the State Rhineland-Palatinate (Approval number: 2020-15342-retrospectiv). The requirement for individual patient consent was deemed not required due to the absence of identifiable data.

This historical cohort study was conducted based on records from the University Medical Centre Mainz internal data acquisition and accounting system (DAQ). The DAQ is based on the data set for external quality control in anesthesia on behalf of the German Society of Anesthesiology and Intensive Care Medicine (Deutsche Gesellschaft für Anästhesiologie und Intensivmedizin, DGAI) and the Association of German Anesthesiologists (Bund Deutscher Anästhesisten, BDA) [12]. Mandatory data input into the DAQ is carried out directly by the responsible anaesthesiologist. Datasets can be subdivided by different medical/operative specialities and airway management techniques. The DAQ was electronically searched for all ATI:FB between January 1, 2019, and December 31, 2020, to retrieve the relating case numbers which allow a manual claim search of associated scanned archived anesthesia records in the electronic hospital information system SAP (SAP Deutschland SE & Co KG, Walldorf, Deutschland). The study only includes data on ATI:FB due to the lack of coding for ATI:VL in the DAQ and its infrequent application in our institution. The data were accessed for research purposes starting from October 2020 after receiving the ethics approval.

Extracted data included demographic patient characteristics: age (years), sex (female/male), BMI (kg/m^2^), ASA status (I,II,III,IV), medical/operative speciality, priority of procedure (elective, urgent, emergency) and median time required for intubation using ATI:FB in minutes. The median time required for intubation was defined as the time elapsed from the patient entering the operation room until release for surgery.

Data extracted from scanned archived anesthesia records included detailed procedural information of the ATI:FB (overall success, route (nasal or oral), experience of anesthesiologist, sedation technique and indication). Complications documented in free-text form on the anesthesia records were retrospectively categorized during analysis into the following groups: technical difficulties (Tube or endoscope replacement with a smaller size due to obstacles in the glottis or tumor area), agitated patient, obstruction of visibility due to mucous secretion, and unspecified complications. Failed ATI:FB cases were excluded from complications and subjected to separate analyses. Authors had access to participant information that could potentially identify individuals, but complete anonymization was conducted prior to analysis to ensure privacy and confidentiality.

### Statistical analysis

Secondary ATI:FB after failure of other airway management techniques and with incomplete datasets were excluded from the analysis. Patient and ATI:FB characteristics were described as absolute and relative numbers and medians with interquartile ranges (IQR).

We used logistic regression to assess the joint impact of route, sedation technique, indication, priority of procedure, experience of anesthesiologist, BMI and medical/operative speciality on the occurrence of complications. In the text we provide p-values from the Likelihood Ratio Chi Squared which tests the hypothesis that all regression coefficient pertaining to a categorical variable are zero. I. e. this test examines the null hypothesis of no association at all between the outcome and a factor. Fig 2 illustrates odds ratios with 95 % confidence intervals when comparing a specific outcome to a reference category and corresponding p-values for the comparison to the reference category. Overall p-values for each category are displayed in the category headings. Reference categories were chosen based on either the category with the lowest expected risk or, in the case of medical/operative speciality and indication, the largest subgroup.

We used linear regression with log(time) as dependent variable to assess the joint impact of the same variable as above on time to perform ATI:FB. The same reference categories were used as in the logistic regression model. P-values of the F-test, which tests the null hypothesis of no association at all between time to perform ATI:FB and a factor, are given in the text. Fig 3 displays marginal mean estimates with 95 % confidence intervals for time to perform ATI:FB (after back-transformation to the original scale). Again, p-values are presented for the comparison of specific categories to the reference category, overall p-values for each category are displayed in the category heading. All variables were included in both regression models simultaneously.

The significance level was set to α = 0.05. Statistical analysis was performed using SAS 9.4.

## Results

**Fig 1:**
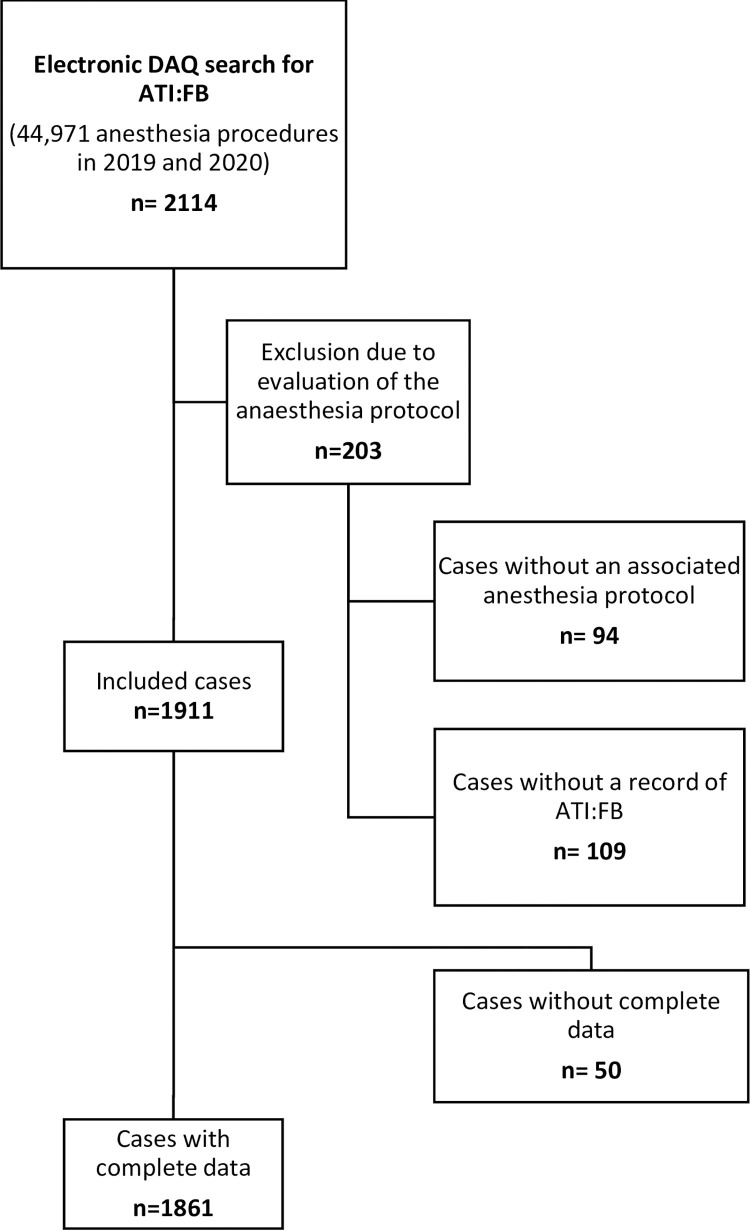
Search strategy flow chart: Electric DAQ (internal data acquisition and accounting system) search for ATI:FB (awake tracheal intubation using flexible bronchoscopy) and manual review of the associated anesthesia records.

A total of 44,971 anesthesia procedures in 2019 and 2020 were electronically searched in the DAQ for ATI:FB, and 2.114 cases of ATI:FB cases were identified. Of these, 94 cases without an associated anesthesia record (separately archived in the electronic hospital information system SAP) were excluded, just like 109 cases in which the manual review of the anesthesia record indicated that it did not involve an ATI:FB procedure (Fig 1). Therefore, 1,911 ATI:FB performed from January 1, 2019, to December 31, 2020, were evaluated, accounting for 4.3 % of all airway management procedures (oral intubation, nasal intubation, mask ventilation [without additional airway intervention], supraglottic airway device, tracheal cannula, double lumen intubation, jet ventilation). 1861 cases with complete data were included in the regression analyses.

### Characteristics of study population

As shown in Table 1, the median (IQR, [range]) age of the patients was 62 (50–72 [<1–98]) years and BMI was 25.5 (22.3–29.5 [13.1–68.9]) kg/m^2^. 22.7 % of the patients undergoing ATI:FB were obese (433/1,911), with obesity classes 1, 2, 3 occurring in 247 (12.9 %), 96 (5.0 %) and 90 (4.7 %) patients, respectively. 34.7 % (664) of the ATI:FB were performed in female patients and 65.3 % (1,247) in male patients. Most of the patients undergoing ATI:FB were classified as ASA III (51 %), followed by ASA II (39 %), ASA I (5 %), and ASA IV (5 %).

**Table 1:** Characteristics of study population and procedural characteristics of awake tracheal intubation using flexible bronchoscopy ATI:FB. Absolute numbers and percentages. Age (years) and body mass index (BMI) are reported as medians and (IQR, [range]).

**Characteristics of study population**	
Age (years)	62 (50–72 [<1–98])
BMI (kg/m^2^)	25.5 (22.3–29.5 [13.1–68.9])
Obesity class	
1	247 (12.9 %)
2	96 (5.0 %)
3	90 (4.7 %)
Sex	
Female	664 (34.7%)
Male	1,247 (65.3%)
ASA status	
I	96 (5%)
II	745 (39%)
III	974 (51%)
IV	96 (5%)
**Procedural characteristics of ATI.FB**	
Priority of procedure	
Elective	1,642 (85%)
Urgent	184 (9.6%)
Emergency	103 (5.4%)
Route	
Nasal	803 (42%)
Oral	961 (50.3%)
Change of route	2 (0.1%)
Not documented	145 (7.6%)
Experience of anesthesiologist	
Less than 1 year	574 (30%)
1 to 5 years	600 (31.4%)
More than 5 years	737 (28.6%)
Sedation technique	
Sufentanil	1,132 (59.2%)
Sufentanil + etomidate	746 (39%)
Sufentanil + ketamine	24 (1.3%)
Remifentanil	9 (0.5%)
Indication	
Oropharyngeal neoplasia	919 (48.1%)
Oropharyngeal inflammation	339 (17.8%)
Suspected difficult airway	280 (14.7%)
Maxillofacial fractures	165 (8.6%)
Cervical spine fractures	104 (5.4%)
Known difficult airway	92 (4.8%)
Other indications	12 (0.6%)

Patients in dental, oral and maxillofacial surgery accounted for 888 (46.5 %) ATI:FB and 733 (38.4 %) in otorhinolaryngology. The remaining ATI:FB were performed in other specialties (Table 2).

**Table 2:** Frequency of awake tracheal intubation using flexible bronchoscopy (ATI:FB) by medical/operative specialty, indication, and complication.

medical/operative specialities	Number of ATI:FB 2019 and 2020 n = 1,911		3 most frequent indications for ATI:FB			Complications		Failed ATI:FB
	n = 1911	%	1. n	2. n	3. n	n	%	n
**dental, oral, and maxillofacial surgery**	888	46.5	oropharyngeal neoplasia 323	oropharyngeal inflammation 251	maxillofacial fractures *163*	*36*	*4*.*1*	*1*
**otorhinolaryngology**	733	38.4	oropharyngeal neoplasia 567	oropharyngeal inflammation 81	known difficult airway 53	47	6.4	3
**neurosurgery**	85	4.4	cervical spine fractures 49	other suspected difficult airway 19	known difficult airway 12	4	4.7	1
**traumatology**	67	3.5	cervical spine fractures 44	other suspected difficult airway 14	known difficult airway 8	6	9.0	0
**ophthalmology**	52	2.7	other suspected difficult airway 34	known difficult airway 11	oropharyngeal neoplasia 4	6	11.5	1
**general surgery**	33	1.7	other suspected difficult airway 14	oropharyngeal neoplasia 9	oropharyngeal inflammation 4	1	3.0	0
**orthopedics**	17	0.9	cervical spine fractures 9	other suspected difficult airway 6	known difficult airway 2	0	0	1
**urology**	10	0.5	other suspected difficult airway 4	known difficult airway 3	oropharyngeal neoplasia 3	1	10.0	0
**gynaecology**	8	0.4	known difficult airway 3	oropharyngeal neoplasia 3	other suspected difficult airway 2	0	0	1
**medical diagnostics**	8	0.4	other suspected difficult airway 6	oropharyngeal neoplasia 2	-	3	37.5	0
**cardiac and thoracic surgery**	6	0.3	other suspected difficult airway 4	oropharyngeal neoplasia 1	known difficult airway 1	0	0	0
**paediatric surgery**	4	0.2	oropharyngeal neoplasia 3	other suspected difficult airway 1		0	0	0

### Procedural characteristics of ATI:FB

As shown in Table 1, 85 % of ATI:FB (n = 1,624) were performed as an elective procedure, 184 (9.6 %) were urgent and 103 (5.4 %) were performed as an emergency. In 961 (50.3 %) ATI:FB oral route was selected and in 803 (42.0 %) nasal routes, with the residual 145 (7.6 %) cases being not documented and 2 (0.1 %) with change of route. 574 (30.0 %) ATI:FB were performed by anesthesia residents with less than 1 year experience. An anesthesia resident with 1 to 5 years of experience performed ATI:FB in 600 (31.4 %) cases. 737 (38.6 %) ATI:FB were performed by anesthesia consultants or residents with more than 5 years of experience.

In 1,132 (59.2 %) ATI:FB an opioid-only sedation technique with sufentanil was used and a combination of sufentanil and etomidate in 746 (39.0 %) patients. Other techniques included a combination of sufentanil and ketamine in 24 (1.3 %) and remifentanil infusion in 9 (0.5 %) ATI:FB.

The most common indication for ATI:FB was oropharyngeal neoplasia in 919 (48.1 %) patients, followed by oropharyngeal inflammation in 339 (17.8 %) cases. Other suspected difficult airway was the indication in 280 (14.7 %) cases, maxillofacial fractures in 165 (8.6 %) cases, cervical spine fractures in 104 (5.4 %) cases, known difficult airway in 92 (4.8 %) cases, and other indications in 12 (0.6 %) cases.

### Overall success rate and analysis of failed cases

The overall success rate of ATI:FB was 99.6 %. Table 3 lists the eight failed cases in which airway management could not be achieved by ATI:FB alone. No cases were cancelled or deferred after a failed ATI:FB. In two of the eight cases, open surgical tracheotomy was successfully performed by an ENT (ear, nose and throat) surgeon.

**Table 3:** Indication, type of failure, reason for failure and management of eight cases of failed awake tracheal intubation using flexible bronchoscopy (ATI:FB).

Case	Indication	Type of failure	Reason for failure	Management
1	oropharyngeal inflammation	oversedation, apnoea	autistic and obese patient	induction of anesthesia, then successful mask bag ventilation and direct laryngoscopy #3 and intubation
2	other suspected difficult airway (Mallampati 3, questionable thyreomental distance < 6 cm, limited extension)	insufficient sedation	inexperienced anesthesiologist	induction of anesthesia, then uncomplicated video laryngoscopy (McGrath blade size 4) and intubation
3	oropharyngeal neoplasia (hypopharyngeal malignoma)	insufficient sedation	unknown	induction of anesthesia, then uncomplicated video laryngoscopy (C-MAC) and intubation
4	other suspected difficult airway (Mallampati 3, questionable thyreomental distance limited extension)	insufficient local anesthesia	allergy against local anesthetics	induction of anesthesia, then uncomplicated video laryngoscopy (C-MAC D-Blade) and intubation
5	other suspected difficult airway (Mallampati 4, questionable thyreomental distance limited extension)	impossible progression with tube	laryngospasm	induction of anesthesia, then uncomplicated video laryngoscopy (McGrath X blade) and intubation
6	known difficult airway	impossible progression with tube	tight glottis	addition of video laryngoscopy in the awake patient (C-MAC D-Blade)
7	oropharyngeal neoplasia (laryngeal malignoma)	unknown	tumour mass	surgical tracheotomy in the awake patient (performed by an ENT surgeon)
8	oropharyngeal neoplasia (thyroid carcinoma)	impossible progression with tube	tumour mass	successful mask bag ventilation after induction of anesthesia, then surgical trachea-tomy (performed by an ENT surgeon)

### Safety of ATI:FB

#### Complications of ATI:FB and influencing factors

Overall, there were 104 (5.4 %) ATI:FB with complications, of which 36 (34.6 %) were not further specified, 25 (24.0 %) were technical difficulties (Tube or endoscope replacement with a smaller size due to obstacles in the glottis or tumor area), 26 (25.0 %) occurred in agitated patients, 17 (16,4 %) were caused by obstruction of visibility due to mucous secretion secretions.

**Fig 2:**
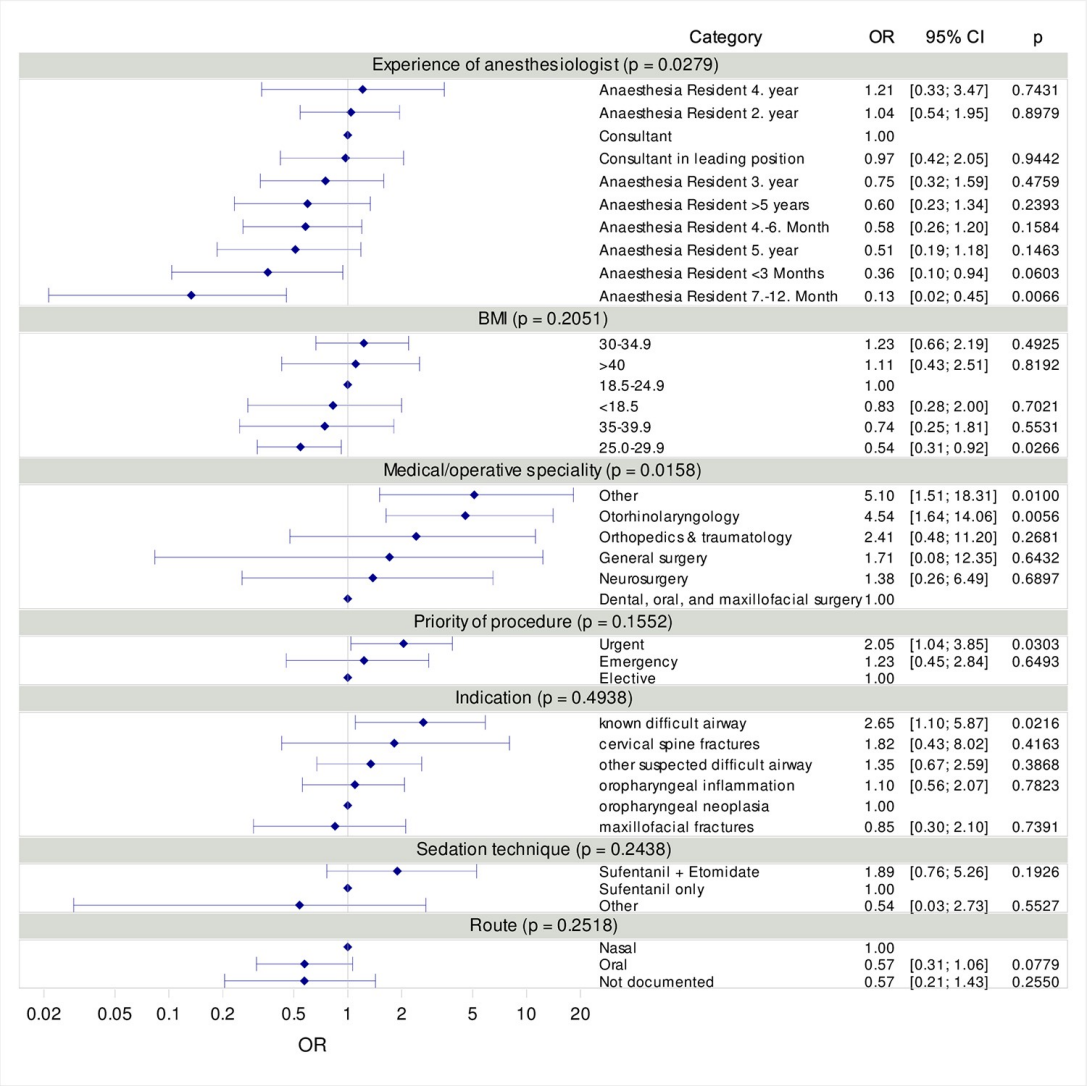
Odds ratios with 95 % confidence intervals when comparing a specific outcome to a reference category and corresponding p-values for the comparison to the reference category. Overall p-values for each category are displayed in the category headings. Reference categories have OR = 1 and no confidence interval.

In a multivariable logistic regression model, the type of medical/operative speciality (p = 0.0158 for the overall test) and experience of anesthesiologist were the only factors (p = 0.0279 for the overall test) with a significant impact on the risk of complications. As shown in Fig 2, compared to dental, oral and maxillofacial surgery, complications were more likely to occur in all other types of medical/operative speciality. The strongest effect was observed in the group of “other” type of medical/operative specialties with OR = 5.10 (95 % CI [1.51; 18.31] and in otorhinolaryngology (OR = 4.54, 95 % CI [1.64; 14.06]).

#### Required time to perform ATI:FB and influencing factors

The median time required for ATI:FB was 16 min [IQR: 11 to 23]. ATI:FB with the indication of a cervical spine fracture took the longest with a median of 24 min [IQR: 17.5 to 35], whereas median time required for ATI:FB with the indication of an inflammation had a median duration of 15 min [IQR: 11 to 20].

**Fig 3:**
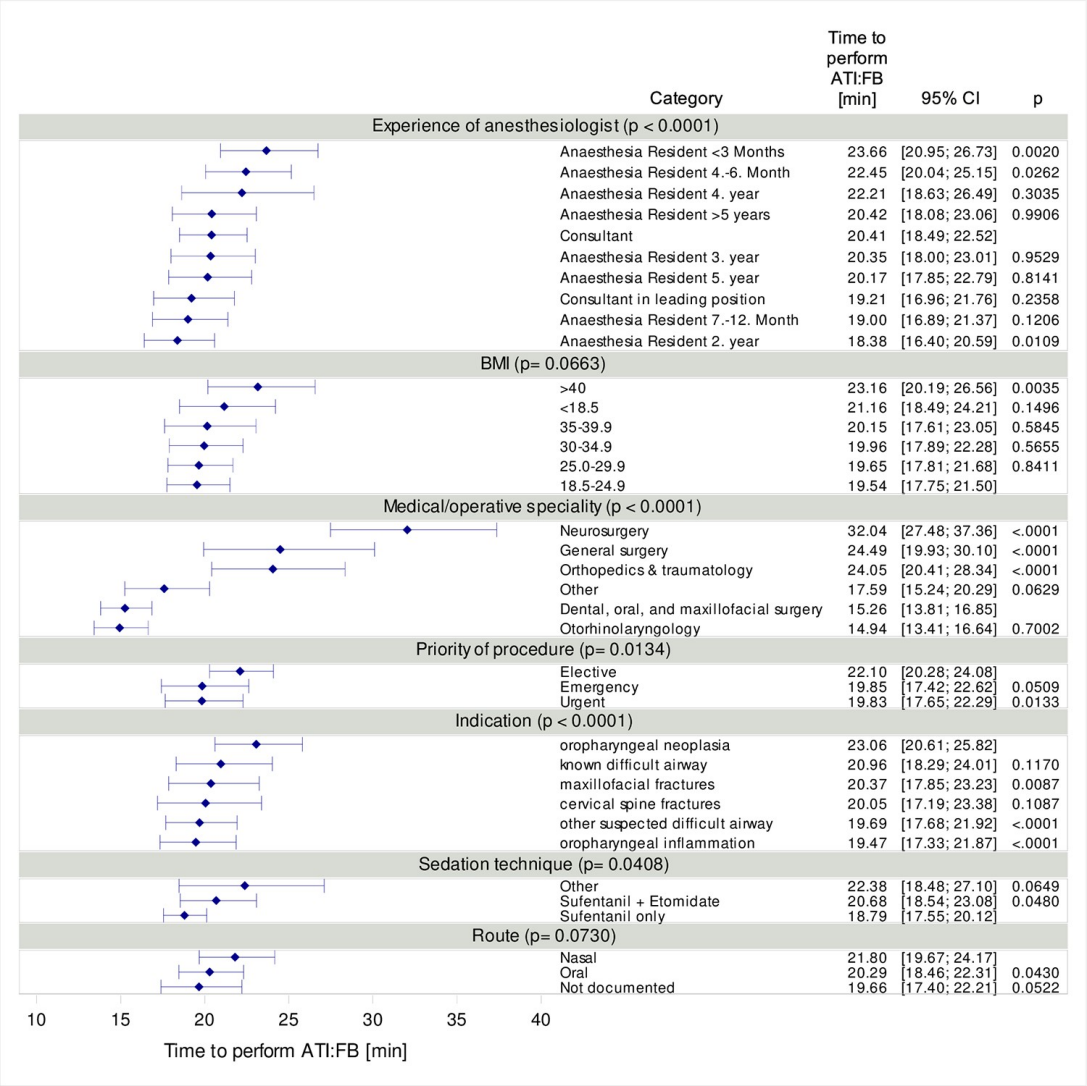
Marginal mean estimates of time to perform an awake tracheal intubation using flexible bronchoscopy (ATI:FB) adjusted for all covariates in the model. Explanatory variables were included in multivariable linear regression model for log(time). Results were back transformed to linear time scale. Overall p-values for each category are displayed in the category headings.

In a multiple linear regression model, significant effects on median time required for intubation using ATI:FB were found for indication (p < 0.0001), level of experience (p < 0.0001), sedation technique (p = 0.0408), priority of procedure (p = 0.0134) and medical/operative specialty (p < 0.0001), while BMI (p = 0.0663) and route (p = 0.0730) did not show significant effects (Fig 3).

## Discussion

This historical study is the only retrospective analysis of contemporary awake intubation practices in Germany. The results indicate that when incorporated into routine airway management, ATI:FB proves to be both effective and safe. In our tertiary institution, we demonstrated that ATI:FB constituted a proportion of 4.3 % of all airway management procedures. Our center achieved a remarkably high success rate for ATI:FB, with only 0.4 % of cases resulting in failure and no procedure-related deaths observed. As anticipated, the majority of cases involved dental, oral, maxillofacial surgery, and otorhinolaryngological procedures, with the most frequent indication being oropharyngeal neoplasia. These elective procedures predominantly opted for an oral route, with one-third performed by an anesthesiologist with less than one year of experience.

The incidence of ATI:FB in our center is notably higher than in previous studies, despite the widespread availability of modern equipment, such as videolaryngoscopes. Joseph et al. from the USA report 0.8 % of all tracheal intubations [5] and Law et al. from Canada 1.06 % [3]. El-Boghdadly et al. from the UK reported a proportion of 1.71 % ATI from all general anaesthetics administered [6]. In this context, it is pertinent to highlight that our ATI:FB ratio of 4.3 % in contrast to data from Joseph and Law is derived from all airway management procedures rather than being limited to tracheal intubations alone. We consistently opt for ATI in patients with changes in the larynx, pharynx, and trachea, considering it a vital aspect of comprehensive diagnostics. This approach is particularly emphasized during panendoscopies in otorhinolaryngology and dental, oral, and maxillofacial surgery procedures. Furthermore, we apply ATI to patients exhibiting multiple predictors for challenging mask ventilation or laryngoscopy, such as a higher Mallampati score, specific dental considerations, or those with a beard. We have been placing a high value on ATI:FB training for decades. As for any manual activity, a solid education and regular training are indispensable prerequisites. In our centre, our residents learn this technique in the first year. To guarantee a sufficiently high number of cases, we set the indication for ATI:FB very generously, because this allows us to train all residents, ensure the required number of 25 ATI to become a specialist, and also ensure that all assisting staff maintain the technical skills of the procedure. Additional solutions involving simulators or rotations in otorhinolaryngology specialties may contribute to training the intubation skills necessary for ATI. Nevertheless, ‘hands-on’ training under experienced guidance with real patients is essential for mastering aspects such as peri-procedure oxygenation, topical anesthesia, and sedation techniques (4). ATI:FB requires a considerable level of expertise and training to avoid complications and reduce failure rates. Demographic changes and economic pressures in German hospitals contribute to reduced education. Additionally, German anesthesiologists often bear external training costs. Quality management efforts, like certificate workshops, aim to ensure practice quality and guideline-appropriate approaches, emphasizing the need for comprehensive initiatives to address training gaps and resource challenges in ATI:FB [7].

Based on our previous findings, the use of ATI:FB in our institution has remained stable over decades, signifying its integration into routine airway management [10]. In contrast, a recent study, encompassing 692 cases of ATI between 2014 and 2020, revealed a significant decrease of approximately 50 % (4). Originally not focused on reasons for a decrease in ATI, the study provides a potential explanation for the widespread belief that ATI is declining. Increasing comfort and trust among clinicians in using videolaryngoscopy to manage anticipated difficult airways after the induction of general anesthesia might be one factor. We share these insights with our Canadian colleagues. Additionally, we align with the thoughts of our American counterparts, including Aibek E. Mirrakhimov et al. [13] who emphasize the importance of high-fidelity simulation and, in our view, particularly stress the significance of deliberate practice, which we actively incorporate into our approach.

The high success rate of ATI.FB at 99,6 % aligns with previously reported success rates [3, 5, 6]. In a recent Norwegian retrospective study involving 833 ATI:FB (0.92 % of all intubations) 3.5  % resulted in failure [14], attributed to low procedural volume per anesthesiologist and exclusive performance of ATI:FB in difficult airway scenarios. Approximately two-thirds of the failed ATI:FB were salvaged with direct and videolaryngoscopy after induction of anesthesia, while one-third required surgical access. These findings are consistent with our data and underscore the significance of these techniques in managing the difficult airway.

The complication rate in our study was 5.4 % of all ATI:FB. Due to varying definitions of complications in other studies, which, in part, included minor complications such as multiple intubation attempts or cough during intubation–incidents not routinely reported by us–we opted not to engage in a comparison of the percentage distributions. The nature of documented complications in our study primarily revolves around technical difficulties (24 %), mirroring the observations in Joseph’s study, where ATI failures were most frequently attributed to the inability to pass the endotracheal tube over the fiberscope (5). This was followed by the challenge of managing agitated patients (25 %), a complication related to cough or gag reflex during intubation also mentioned by Law (3).

This encompasses the two aspects of sedation technique and topical anesthesia within the overall concept of ATI. Notably in our study, the complication arising from the challenge of visibility obstruction due to mucous secretions is observed to be 16.4 % and therefore higher than in other studies. This increased mucosal secretion may be attributed to the fact that the application of anti-salivation, which could potentially prevent this complication, is not routinely practiced in our center. This breakdown of complication analysis provides valuable insights into the challenges encountered during ATI, highlighting areas for potential improvement and targeted interventions to enhance the safety of ATI.

Multivariable logistic regression identified the type of medical/operative specialty and anesthesiologist experience as significant factors influencing complication risk. However, it should be noted that the correlation between increasing experience and decreasing risk was not included in the logistic regression used to assess the joint impact of the influencing factors on complications. Further, when considering the odds ratios in Fig 2, it’s important to note that the individual influencing factors are not independent. The elevated risk of complications (OR = 5.10) in the ‘others’ category may be attributed, for instance, to the low incidence in these unfamiliar settings such as medical diagnostics indicating a less routine environment (Table 2). In contrast, the increased rate of complications (OR = 4.54) in otolaryngology may be linked to the fact that our least experienced colleagues are often deployed in this specialty. Additionally, the presence of large tumors that obstruct airways and may bleed, characteristic of this field, poses a particular challenge during procedures. This emphasizes the importance of tailoring training programs and support measures to address specific challenges associated with different specialties.

The median time required for intubation using ATI:FB was 16 min in our study. Joseph et al. reported a median time of 24 minutes for successful ATI:FB in awake patients. The time is defined as the duration from the patient entering the operating room to the time of intubation. In our study, the endpoint for measuring time is defined as the release into the operating room after completing intubation and all other necessary procedures. It is assumed, however, that these additional procedures, such as a second IV or arterial line, are only necessary in complex, individual cases. We assume that, due to the high case volume, these isolated instances do not significantly impact the median time required for ATI:FB. The median time for ATI:VL (n = 22) in Joseph’s study was also 24 minutes, consistent with data from Denmark, which similarly showed no time difference between ATI:FB and ATI:VL [15] However, having an estimation of the additional time required for ATI:FB is helpful for managing expectations. In elective surgeries, decisions about airway management, particularly for difficult airways, should not be influenced by perceived time pressure or airway manager discomfort with performing ATI. According to updated recommendations from the Canadian Airway Focus Group, seeking assistance from a more experienced colleague in performing ATI is recommended [16].

The limitations include the retrospective nature of the data analysis, which is associated with a risk of bias and a potentially diminished transferability of the data into practice. Underreporting of complications caused by subjective assessment may have affected the reported incidence rates. Moreover, there was no direct control group with alternative intubation techniques to conclude on the safety and complication rate in comparison to other anesthesia procedures. As ATI:FB is often replaced by video laryngoscopy used after the induction of general anesthesia, a comparison between the frequency of ATI:FB and video laryngoscopy after induction of anesthesia in our centre would be of value, yet the DAQ does not map data on video laryngoscopy after induction of anesthesia nor on ATI:VL. Our results show that in 5 out of 8 failures related to ATI:FB, airway management was eventually accomplished using video laryngoscopy after induction of anesthesia. Moreover, the fact that our data reflects the situation in a single institution must be acknowledged as a limitation. In terms of the linear regression model, the explanatory covariates are not independent from each other and therefore the estimated marginal means sometimes differ from the naïve descriptive measures. In other words, the figures estimated as part of the linear regression may not be identical with the calculated descriptive figures because the latter do not take into account other variables that could affect the figure.

## Conclusion

ATI:FB is a successful and safe technique, characterized by a low complication rate and a manageable time to perform. A modifiable factor contributing to enhanced safety is the experience of the anesthesiologist. Consequently, ATI:FB should be an integral component of routine airway management.

Furthermore, early incorporation of ATI:FB into anesthesiology training is crucial for the safe handling of difficult airways. Anesthesiologists should be actively encouraged to regularly perform ATI to acquire and sustain valuable experience in this technique.

## Supporting information

S1 Checklist STROBE statement—checklist of items that should be included in reports of observational studies. (PDF)

S1 Data (SAV)
