# Supplementary material for: Awake tracheal intubation in routine airway management: A retrospective analysis in a tertiary centre
Source: PLoS One. 2024 Mar 1;19(3):e0299071. doi: 10.1371/journal.pone.0299071 (PMC10906896; doi:10.1371/journal.pone.0299071)
Supplement: S1 Checklist — (PDF) [file pone.0299071.s001.pdf]

STROBE Statement—checklist of items that should be included in reports of observational studies

	Item No.	Recommendation	Page No.	Relevant text from manuscript
**Title and abstract**	1	(*a*) Indicate the study's design with a commonly used term in the title or the abstract	1	Awake tracheal intubation in routine airway management: A historical cohort study in a tertiary centre
		(*b*) Provide in the abstract an informative and balanced summary of what was done and what was found	3	Records of the internal data acquisition system and archived anesthesia protocols were screened for ATI:FB conducted in 2019 and 2020 and the specifics of the procedures assessed. 4.3 % (n = 1,911) of all airway management procedures were ATI:FB, with most ATI:FB conducted in the departments of dental, oral and maxillofacial surgery (46.5 %) and otorhinolaryngology (38.4 %). The success rate of ATI:FB was 99.6 %. Complication rates were dependant on the type of medical specialty and the experience of the anesthesiologist, with the highest odds ratio noted for otorhinolaryngology (OR = 4.35, 95% CI [1.60; 13.11]).

				The median time required for ATI:FB was 16 min [IQR: 11 to 23], with significant effects on duration observed for indication ($p < 0.0001$), level of experience ($p = 0.0001$), sedation technique ($p = 0.0408$), type of procedure ($p = 0.0134$), and clinic/surgery ($p < 0.0001$). The time for ATI:FB differed significantly according to the experience of the anesthesiologist ($p < 0.0001$).
**Introduction**				
Background/rationale	2	Explain the scientific background and rationale for the investigation being reported	4	The aim of our retrospective cohort study was to assess the quality and potential problems associated with the performance of ATI at our tertiary center over a 2-year period and describe current indications, success and failure rates, complications and the required time to perform this technique depending on the indication, patient characteristics and anesthesiologist training level.
Objectives	3	State specific objectives, including any prespecified hypotheses	4	Our hypothesis is that ATI represents an extremely safe method for the expected difficult airway. If we are able

				to confirm this hypothesis the threshold for anesthesiologists to perform ATI should be lowered by eliminating the above mentioned biases combined with good training.
**Methods**				
Study design	4	Present key elements of study design early in the paper	5	This historical cohort study was conducted based on records from the University Medical Centre Mainz internal data acquisition and accounting system (DAQ).
Setting	5	Describe the setting, locations, and relevant dates, including periods of recruitment, exposure, follow-up, and data collection	5	Datasets can be subdivided by different medical/operative specialities and airway management techniques. The DAQ was electronically searched for all ATI:FB between January 1, 2019 and December 31, 2020 to retrieve the relating case numbers which allow a manual claim search of associated scanned archived anesthesia protocols in the electronic hospital information system SAP (SAP Deutschland SE & Co KG, Walldorf, Deutscland).<br>Extracted data included demographic patient

				characteristics: number of patients per specialisation, age (years), sex (female/male), ASA status (I,II,III,IV), airway evaluation data (Mallampati category, neck reclination, thyromental distance) and priority of procedure (elective, urgent, emergency), and time required until successful intubation in minutes. This time was defined as the time elapsed from the patient entering the operation room until release for surgery.<br>Data extracted from scanned archived anesthesia protocols included detailed procedural information (success/failure of the ATI:FB, route of ATI:FB (nasal or oral), level of experience of intubator, sedation technique, indication for the ATI:FB).
Participants	6	(*a*) *Cohort study*—Give the eligibility criteria, and the sources and methods of selection of participants. Describe methods of follow-up<br>*Case-control study*—Give the eligibility criteria, and the sources and methods of case ascertainment and control selection. Give the rationale for the choice of cases and controls<br>*Cross-sectional study*—Give the eligibility criteria, and the sources and methods of selection of participants	5	The DAQ was electronically searched for all ATI:FB between January 1, 2019 and December 31, 2020 to retrieve the relating case numbers which allow a manual claim search of associated scanned archived

				anesthesia protocols in the electronic hospital information system SAP (SAP Deutschland SE & Co KG, Walldorf, Deutscland).
		(*b*) *Cohort study*—For matched studies, give matching criteria and number of exposed and unexposed<br>*Case-control study*—For matched studies, give matching criteria and the number of controls per case		
Variables	7	Clearly define all outcomes, exposures, predictors, potential confounders, and effect modifiers. Give diagnostic criteria, if applicable	5	Not applicable
Data sources/ measurement	8*	For each variable of interest, give sources of data and details of methods of assessment (measurement). Describe comparability of assessment methods if there is more than one group	*5*	*records from the University Medical Centre Mainz internal data acquisition and accounting system (DAQ) and manual claim search of associated scanned archived anesthesia protocols in the electronic hospital information system SAP*
Bias	9	Describe any efforts to address potential sources of bias	5	Not applicable
Study size	10	Explain how the study size was arrived at	5	The DAQ was electronically searched for all ATI:FB between January 1, 2019 and December 31, 2020.

Continued on next page

Quantitative variables	11	Explain how quantitative variables were handled in the analyses. If applicable, describe which groupings were chosen and why	5	Not applicable
Statistical methods	12	(*a*) Describe all statistical methods, including those used to control for confounding	5+6	Statistical Analysis<br>Secondary ATI:FB after failure of other airway management techniques, with additional extensive complex procedures, and with incomplete datasets were excluded from the analysis. Patient and ATI:FB characteristics were described as absolute and relative numbers and medians with interquartile ranges (IQR). Variables with a potential impact on time to perform ATI:FB and probability of complication were assessed in regression models. R^2 was used to describe the proportion of variance explained by the linear regression model and Nagelkerke's R^2 was used for the logistic regression model. The significance level was set to $\alpha = 0.05$. Statistical analysis was performed using SAS 9.4. and R 4.2.2 (12), specifically the packages relaimpo (13) and rsq (14)
		(*b*) Describe any methods used to examine subgroups and interactions	5	Datasets can be subdivided by different medical/operative specialities and airway management techniques.

			Extracted data included demographic patient characteristics: number of patients per specialisation, age (years), sex (female/male), ASA status (I,II,III,IV), airway evaluation data (Mallampati category, neck reclination, thyromental distance) and priority of procedure (elective, urgent, emergency), and time required until successful intubation in minutes.<br>Data extracted from scanned archived anesthesia protocols included detailed procedural information (success/failure of the ATI:FB, route of ATI:FB (nasal or oral), level of experience of intubator, sedation technique, indication for the ATI:FB).
	(*c*) Explain how missing data were addressed	7	1861 cases with complete data were included in the regression analyses. Incomplete Data was excluded.
	(*d*) *Cohort study*—If applicable, explain how loss to follow-up was addressed<br>*Case-control study*—If applicable, explain how matching of cases and controls was addressed<br>*Cross-sectional study*—If applicable, describe analytical methods taking account of sampling strategy		Not applicable
	(*e*) Describe any sensitivity analyses		Not applicable
**Results**			

Participants	13*	(a) Report numbers of individuals at each stage of study—eg numbers potentially eligible, examined for eligibility, confirmed eligible, included in the study, completing follow-up, and analysed	7	The electronic DAQ search identified 2.114 cases of ATI:FB. Of these, 94 cases without an associated anesthesia protocol were excluded , as were 109 cases without a record of ATI:FB in the anesthesia. Therefore, 1,911 ATI:FB performed from January 1, 2019 to December 31, 2020 were evaluated, accounting for 4.3 % of all airway management procedures. 1861 cases with complete data were included in the regression analyses.
		(b) Give reasons for non-participation at each stage	6	Secondary ATI:FB after failure of other airway management techniques, with additional extensive complex procedures, and with incomplete datasets were excluded from the analysis.
		(c) Consider use of a flow diagram		The numerical data regarding non-participation can be effectively conveyed as continuous prose, thereby rendering a flow chart redundant.
Descriptive data	14*	(a) Give characteristics of study participants (eg demographic, clinical, social) and information on exposures and potential confounders	7	*Characteristics of patients with ATI:FB* The median (IQR, [range]) age of the patients was 62 (50-72 [<1-98]) years and BMI was 25.5 (22.3-29.5 [13.1-68.9]) kg/m^2. 22.7% of the

			patients undergoing ATI:FB were obese (433/1,911), with obesity classes 1, 2, 3 occuring in 247 (12.9%), 96 (5.0%) and 90 (4.7%) patients, respectively. 34.8% (665) of the ATI:FB were performed in female patients and 65.5% (1,251) in male patients. Most of the patients undergoing ATI:FB were classified as ASA III (51%), followed by ASA II (39%), ASA I (5%), and ASA IV (5%).
		(b) Indicate number of participants with missing data for each variable of interest	Not applicable
		(c) *Cohort study*—Summarise follow-up time (eg, average and total amount)	Not applicable
Outcome data	15*	*Cohort study*—Report numbers of outcome events or summary measures over time	
		Case-control study—Report numbers in each exposure category, or summary measures of exposure	
		Cross-sectional study—Report numbers of outcome events or summary measures	
Main results	16	(*a*) Give unadjusted estimates and, if applicable, confounder-adjusted estimates and their precision (eg, 95% confidence interval). Make clear which confounders were adjusted for and why they were included	Not applicable
		(*b*) Report category boundaries when continuous variables were categorized	Not applicable
		(*c*) If relevant, consider translating estimates of relative risk into absolute risk for a meaningful time period	Not relevant

Continued on next page

Other analyses	17	Report other analyses done—eg analyses of subgroups and interactions, and sensitivity analyses	4	Our recent study on the frequency of ATI in Germany revealed a discrepancy between the availability of ATI:FB equipment for German anesthesiologists (> 90%) and their proficiency in the technique (60%) (3). Only half of German anesthesiologists stated that they had performed more than 25 ATI:FBs throughout their career.
**Discussion**				
Key results	18	Summarise key results with reference to study objectives	10	The success rate and safety of ATI:FB have manifested this technique in guidelines for management of the difficult airway, yet alternative airway management procedures are often chosen. In this historical study on anesthesia practice in a tertiary institution, 44,971 anesthesia procedures in 2019 and 2020 were reviewed for the frequency and indication of ATI:FB. It was revealed that 1.911 ATI:FB procedures were conducted within this timeframe, with a proportion of 4.3 % of all airway management procedures. We observed a very high success rate for ATI:FB with only 0.4% failed cases in our centre

				and no procedure-related deaths. As expected, patients receiving dental, oral, maxillofacial surgery and otorhinolaryngological procedures accounted for 84.7% of ATI:FB.
Limitations	19	Discuss limitations of the study, taking into account sources of potential bias or imprecision. Discuss both direction and magnitude of any potential bias	12	The limitations include the retrospective nature of the data analysis that is associated with a risk of bias and a potentially diminished transferability of the data into practice. Underreporting of complications caused by subjective assessment may have affected the reported incidence rates. Moreover, there was no direct control group with alternative intubation techniques to conclude on the safety and complication rate in comparison to other anesthesia procedures. As ATI is often replaced by video laryngoscopy, a comparison between the frequency of ATI and video laryngoscopy in our centre would be of value, yet the DAQ does not map data on awake videolaryngoscopy. In terms of the

				safety of the procedure and in light of the longer observed time requirement, the fact that we cannot control for the SOP conformity of the procedure must be mentioned as a limitation. The time required for the procedure may be subject to errors, as the timestamps do not necessarily represent the procedure, as it could be assumed that the time to perform ATI is shorter than the total time of anesthesia induction. In terms of the linear regression model, the explanatory covariates are not independent from each other and therefore the estimated marginal means sometimes differ from the naïve descriptive measures.
Interpretation	20	Give a cautious overall interpretation of results considering objectives, limitations, multiplicity of analyses, results from similar studies, and other relevant evidence	11+12	Discussion
Generalisability	21	Discuss the generalisability (external validity) of the study results	12	The limitations include the retrospective nature of the data analysis that is associated with a risk of bias and a potentially diminished transferability of the data into practice.
**Other information**				

Funding	22	Give the source of funding and the role of the funders for the present study and, if applicable, for the original study on which the present article is based	Not applicable

*Give information separately for cases and controls in case-control studies and, if applicable, for exposed and unexposed groups in cohort and cross-sectional studies.

Note: An Explanation and Elaboration article discusses each checklist item and gives methodological background and published examples of transparent reporting. The STROBE checklist is best used in conjunction with this article (freely available on the Web sites of PLoS Medicine at http://www.plosmedicine.org/, Annals of Internal Medicine at http://www.annals.org/, and Epidemiology at http://www.epidem.com/). Information on the STROBE Initiative is available at www.strobe-statement.org.
